# Neuromuscular Electrical Stimulation for Sialorrhea in an Elderly Woman With Parkinson’s Disease

**DOI:** 10.7759/cureus.24871

**Published:** 2022-05-10

**Authors:** Yuki Kojima, Yutaka Sakaguchi

**Affiliations:** 1 Anesthesiology, Asahi General Hospital, Asahi, JPN; 2 Dentistry, Sakaguchi Dental Clinic, Chiba, JPN

**Keywords:** home medical care, oral rehabilitation, parkinson's disease, sialorrhea, neuromuscular electrical stimulation

## Abstract

Sialorrhea, a common symptom of Parkinson's disease (PD), is related to reduced oromotor control and autonomic dysfunction. However, neuromuscular electrical stimulation (NMES) helps improve overall swallowing function. We performed NMES for eight weeks in an 84-year-old woman with stage 5 PD and severe sialorrhea. The severity and frequency of drooling improved within one to three weeks of NMES. NMES can be used for patients who have lost the will to be rehabilitated or are unable to control an appropriate rehabilitation load themselves. It may also be useful for patients with multiple complications who are unable to commence new medications or injections.

## Introduction

Sialorrhea (excessive salivation) is a common symptom of Parkinson's disease (PD) and is related to reduced oromotor control and autonomic dysfunction. It is observed in 70-78% of patients with PD and greatly affects the patient’s quality of life [1-4]. This condition causes discomfort, and the spillage of saliva can also lead to aspiration and infection [5]. For patients with mild symptoms, oral rehabilitation such as the use of chewing gum or hard candy to encourage swallowing helps alleviate the symptoms [6], while for those with more severe sialorrhea, botulinum toxin injection in the salivary glands, as well as glycopyrrolate treatment, are effective [7] in alleviating the symptoms [8]. However, these treatments have adverse side effects, which can be challenging for patients who find it difficult to move around and need medical care at home. However, rehabilitation must be continued throughout life and is often mentally distressing. Once rehabilitation is stopped, the patients’ quality of life begins to decline again. Rehabilitation is also difficult for patients with reduced ability to function and for those living alone. Such situations are increasing in an ageing society.

Neuromuscular electrical stimulation (NMES) involves direct stimulation of the muscles to recruit motor units and increase muscle strength. The findings of a meta-analysis of seven trials provided evidence in favour of using NMES for swallowing [9], thereby suggesting its potential as an effective treatment for disordered movement in the head region. NMES is applied at higher frequencies (20-50 Hz) expressly to produce muscle tetany and contraction that can be used for "functional" purposes, and its application has been found in the literature reported as early as 1964 [10]. In sports medicine, NMES has been used for muscle strengthening, maintaining muscle mass and strength during prolonged periods of immobilisation, selective muscle retraining, and controlling oedema.

NMES is widely used in stroke rehabilitation. The electrical stimulation of muscle contraction is synchronised with the intended motion to restore movement after paralysis. Here, we evaluate the potential of using NMES for effective rehabilitation in a patient with sialorrhea.

## Technical report

**Figure 1:**
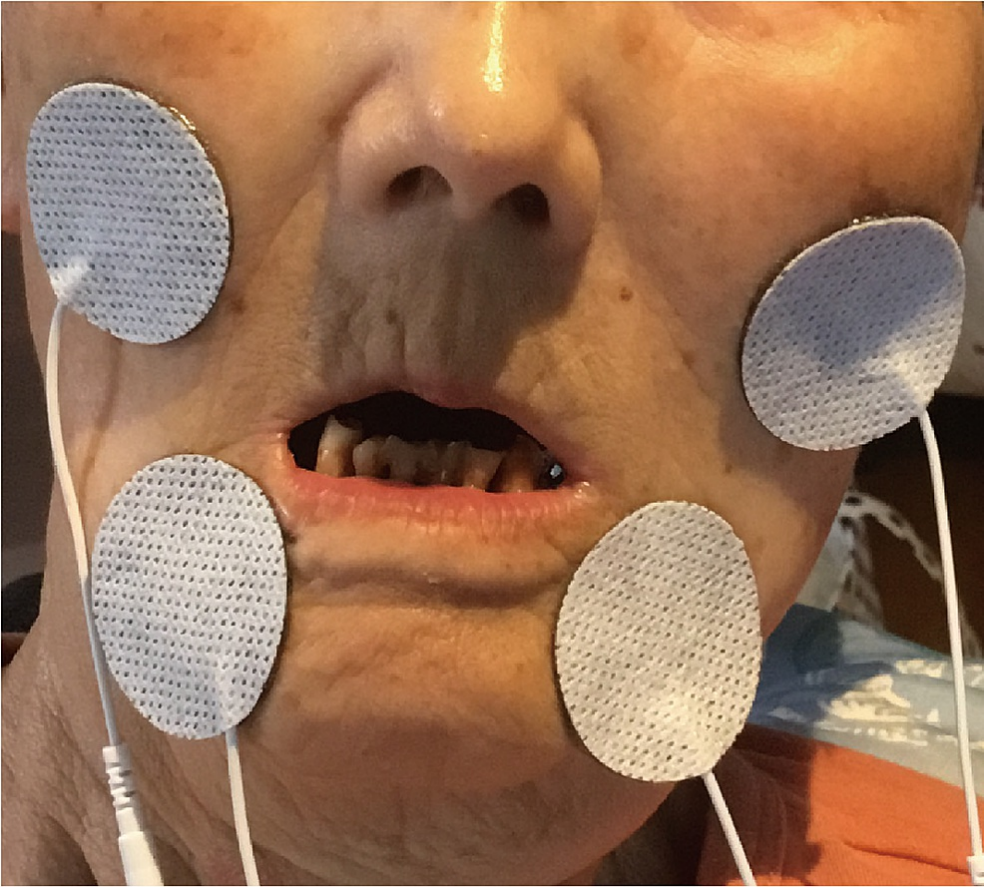
Neuromuscular electrical stimulation unit used to stimulate the orbicularis oris and masseter muscles.

The NMES unit (ExcareDi; Excare Japan, Tokyo, Japan) was set to stimulate the orbicularis oris and masseter muscles (Figure 1). We performed training with NMES on the paralysed side for 30 minutes per day. No other rehabilitation was performed in the facial area during rehabilitation with NMES. The NMES stimulus was compound corrugation with a composite high frequency of 6-9 mA. This rehabilitation can be performed in either the supine or the sitting positions. We instructed the patient’s family on how to apply the NMES pad and instructed them to do it every day. If the patient was sleepy, they were allowed to rehabilitate while sleeping. Before each rehabilitation, the intensity of the stimulus was set so that the patient did not feel any pain although they felt the stimulus.

Case presentation

The patient was an 84-year-old woman with a history of PD and hypertension. According to the Hoehn and Yahr scale, she had stage 5 PD. She used a wheelchair and was unable to live an independent life. Oral rehabilitation was performed five years prior, but its efficacy diminished as the symptoms progressed, and further motivation for rehabilitation declined. About two years prior, the sialorrhea became so severe that it interfered with daily life. The patient had been receiving oral rehabilitation in the form of swallowing training. However, she was unable to undergo rehabilitation for long periods because of fatigue; therefore, continuous oral rehabilitation could not be achieved. The Drooling Severity and Frequency Scale (DSFS) was used to evaluate the patient. According to the baseline DSFS score, which is the sum of the severity and frequency subscores (Table 1), the patient was diagnosed with severe sialorrhea.

**Table 1:** The Drooling Severity and Frequency Scale (DSFS). DSS: Drooling Severity Scale; DFS: Drooling Frequency Scale.

Date	DSS	DFS	Total score
0 week	4	8	12
1 week	3	8	11
2 weeks	3	8	11
3 weeks	3	7	10
4 weeks	3	7	10
5 weeks	2	7	9
6 weeks	2	7	9
7 weeks	2	7	9
8 weeks	2	7	9
9 weeks	1	6	7
10 weeks	2	7	9
11 weeks	1	6	7
12 weeks	2	7	9

The NMES unit was set to stimulate the orbicularis and masseter muscles (Figure 1). We performed training with NMES on the paralyzed side for 30 min per day. No other rehabilitation was performed in the facial area during rehabilitation with NMES. The NMES stimulus was compound corrugation with a composite high frequency of 6-9 mA.

**Figure 2:**
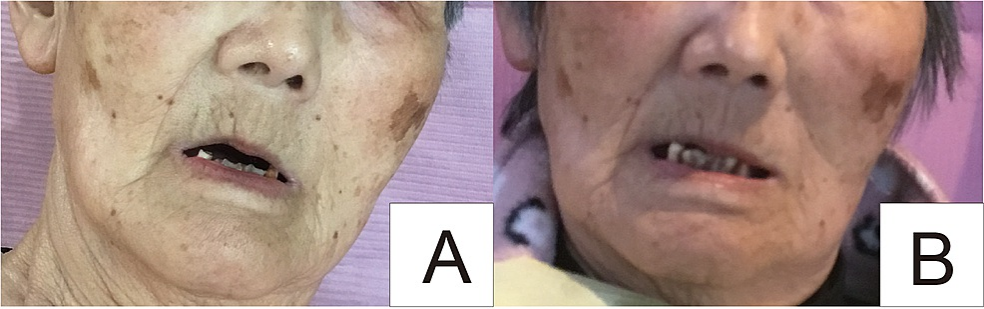
Effect of neuromuscular electrical stimulation on facial muscles. An improvement in the wrinkles of the rear wheels from slight stretching of the facial muscles can be observed. (A) Before treatment. (B) Twelve weeks later.

The severity and frequency of drooling improved after one and three weeks, respectively, of commencing rehabilitation with NMES (Table 1). The severity score was initially 4; it was reduced to 3 after one week and 2 after five weeks. The frequency of drooling was initially eight but became seven after three weeks. No side effects or major complications were observed. The NMES unit was set to stimulate the orbicularis and masseter muscles (Figure 1). Overall, no significant differences were observed in the patient’s facial features before and after eight weeks of treatment (Figures 2A, 2B). The patient was either in the sitting or supine position during treatment, and the procedure was performed for 28 days. It can be determined from the results that the lip closure was incomplete; therefore, it is inferred that stimulation of the orbicularis oris and masseter muscles was useful.

## Discussion

There may be restrictions on the rehabilitation that can be performed due to various factors. Patients with movement disorders such as severe PD make it difficult to perform rehabilitation using their own weight. Patients who are unable to self-manage have difficulty performing home rehabilitation in the absence of caregivers. Mental fatigue makes it impossible to maintain motivation for physical performance [11]. Therefore, it is beneficial for patients to be able to use various methods of rehabilitation. Not only is it effective, but it also enables the patient to feel a sense of accomplishment.

When performing rehabilitation with NMES, it is important to properly position the pad to the target muscle to be rehabilitated to obtain the desired effect. NMES is unlikely to be suitable for patients with electronic devices embedded in their bodies. Therefore, caution is required when using it for patients who have an indwelling pacemaker. During NMES, the facial muscles move involuntarily, which can feel strange until the patient gets accustomed to the treatment. Additionally, if the NMES output is too high, it can affect the teeth and cause pain. For patients with decreased activities of daily living, nutritional management is also very important, along with rehabilitation [12]. In conclusion, NMES can be used to treat patients who have lost the will to be rehabilitated or those who are unable to control their appropriate rehabilitation load themselves. In addition, it may be useful for patients with multiple complications who are unable to commence new medications or injections. In the present case, we studied the rehabilitation of only the jaw muscles. However, further clinical studies are required to evaluate the rehabilitation of other facial muscles, especially to determine the optimal load and treatment duration for positive outcomes.

## Conclusions

The use of NMES may reduce sialorrhea in patients with PD. The rehabilitation effect may give the patient a chance to improve their motivation for rehabilitation. However, further studies are required to evaluate the rehabilitation of other facial muscles, especially to determine the optimal load and treatment duration for positive outcomes. In addition, studies will be needed to determine the long-term effects of NMES to determine the optimal duration of therapy.
